# Feasibility of a new clinical journal club implementation and its association with knowledge, attitudes, and application of evidence-based practice among chiropractic students and trainees: a before-and-after healthcare education improvement study

**DOI:** 10.1186/s12998-023-00494-0

**Published:** 2023-07-24

**Authors:** Melanie Häusler, Rahim Lalji, Léonie Hofstetter, Cesar A. Hincapié

**Affiliations:** 1 EBPI-UWZH Musculoskeletal Epidemiology Research Group, University of Zurich and Balgrist University Hospital, Forchstrasse 340, Zurich, 8008 Switzerland; 2 Department of Chiropractic Medicine, Faculty of Medicine, Balgrist University Hospital, University of Zurich, Zurich, Switzerland; 3 Epidemiology, Biostatistics and Prevention Institute (EBPI), University of Zurich, Zurich, Switzerland; 4 University Spine Centre Zurich (UWZH), Balgrist University Hospital, University of Zurich, Zurich, Switzerland

**Keywords:** Evidence-based practice, Medical education, Chiropractic, Health knowledge, attitudes, practice, Health personnel education, Surveys and questionnaires, Psychometrics

## Abstract

**Background:**

Evidence-based practice (EBP) is the integration of best research evidence with clinical expertise and patients’ values and preferences. Little is known about knowledge, attitudes, and application of EBP among chiropractic students and trainees. Our aims were to (1) examine the feasibility of implementing a new journal club format within a Swiss university chiropractic healthcare education setting, and (2) assess the associations between the new journal club implementation and EBP characteristics among chiropractic students.

**Methods:**

A before-and-after study was conducted through a newly implemented journal club with 5th and 6th year chiropractic students and postgraduate trainees between 1 February 2021 and 31 July 2021. The journal club was developed based on the “community of practice” and “team-based learning” conceptual frameworks. EBP knowledge, attitudes, personal application, and future use, were assessed with a validated questionnaire. We summarised participant characteristics using descriptive statistics, estimated before-and-after EBP total and subscale scores (i.e., knowledge, attitudes, personal application, and future use), and conducted an exploratory subgroup analysis based on journal club attendance (Group A: 3–5 sessions attended; Group B: ≤ 2 sessions attended).

**Results:**

Among 32 eligible students and trainees, 29 participants (mean age 26 years; 79% women) were enrolled: 25 (78%) responded to the pre- and 29 (91%) to the post-assessment surveys. Most (80%) were chiropractic students and 20% were postgraduate trainees. Group A consisted of 12 (41%) and Group B of 17 (59%) participants, respectively. We found reasonable feasibility for the new journal club format and our findings were compatible with no difference in before-and-after EBP scores (median EBP total score before: 72.6 [IQR, 63.7–77.4], and after: 73.4 [IQR, 61.3–78.2]). Exploratory subgroup analyses based on journal club attendance were consistent with our overall findings.

**Conclusion:**

Our study suggests that the newly implemented journal club and embedding chiropractic educational research within the journal club were feasible and acceptable. Small before-and-after differences in the EBP subscale scores for knowledge, attitudes, personal application, and future use were observed in chiropractic students and postgraduate trainees. The small study size and short timeframe during a single semester limit potential inferences.

**Supplementary Information:**

The online version contains supplementary material available at 10.1186/s12998-023-00494-0.

## Background

Journal clubs have been used as a teaching format within academic medicine for almost 150 years [1]. Initially used to keep up-to-date with relevant health literature, journal clubs are now considered a viable format to enhance critical appraisal skills and promote evidence-based practice (EBP) in semi-structured learning environments [2]. EBP has been conceptualised as the integration of the best research evidence with clinical expertise and patient preferences for the purpose of providing care that reflects the interests, values, and needs of the patient [3]. Courses and training have been implemented internationally into undergraduate medical curricula and medical residency programs with the aim of instilling lifelong learning and evidence-based healthcare practice [4, 5]. There remains, however, a lack of consensus on how to best integrate EBP into undergraduate medical curricula [6, 7]. Most of the research has focused on postgraduate education, and reports investigating the undergraduate learning environment are scarce. In one example, Green and colleagues used a modified journal club format that included both didactic and practical elements (use of critical appraisal tools, letter-to-editor writing projects) and found that this format provided a means by which chiropractic students may improve their critical appraisal skills [8].

Within the department of chiropractic medicine at Balgrist University Hospital, journal clubs had been used to facilitate exchange between researchers, clinicians, and students. While it was routine for experienced researchers to lead discussions and critically appraise the literature, chiropractic students with clinical interests and less research experience often found this format challenging. Common student feedback, for example, suggested that some students felt isolated and not sufficiently supported during the journal club preparation phase and found the journal presentations intimidating (done as individual student presenters in a traditional unidirectional didactic lecture format in front of an audience of experienced non-clinician researchers). Similar experiences have been reported in other academic health institutions, with reports of medical journal club content lacking clinical relevance and containing an unnecessarily large focus on biostatistics [9, 10]. Given this experience, a new journal club format was created based on the conceptual frameworks of ‘community of practice’ and ‘team-based learning’ [11], and following established recommendations on how to run an effective journal club [12].

To examine the implementation of this new journal club format, we followed Kirkpatrick’s four-level model — a widely recognized method of evaluation of education programs [13]. The objectives of this study were to (1) assess the feasibility of the new journal club implementation, and (2) estimate associations between the new journal club implementation and EBP characteristics (i.e., knowledge, attitudes, and application of EBP) among chiropractic medicine students and trainees over one academic semester.

## Methods

### Setting

The department of chiropractic medicine is integrated within Balgrist University Hospital, a leading musculoskeletal (MSK) specialized hospital affiliated with the University of Zurich, in Switzerland. Chiropractic medicine students at the University of Zurich complete a six-year curriculum. To fulfil the academic requirements to become a chiropractor, students must first complete a three-year bachelor’s program in human medicine and a subsequent three-year master’s program in chiropractic medicine. During the last year, students spend 6 months in the outpatient chiropractic polyclinic [14] and 6 months rotating through other specialties (e.g., orthopaedics, rheumatology, neurology, radiology, and sports medicine).

### Study design

We carried out a before-and-after study with 5th and 6th year chiropractic students and postgraduate residents during the Spring 2021 semester, between 1 and 2021, and 31 July 2021. All 5th year chiropractic students of the chiropractic masters study program, and 6th year chiropractic students (i.e., “underassistants” within the Faculty of Medicine, University of Zurich), as well as postgraduate residents and PhD students, from the chiropractic polyclinic and research, respectively, were eligible and invited to participate.

Our study was reported according to the SQUIRE-EDU (Standards for QUality Improvement Reporting Excellence in Education) guideline [15] (see Additional file 1 for SQUIRE-EDU checklist). The local independent research ethics committee of Canton Zurich deemed that ethical approval was not required for this healthcare education feasibility study of Swiss chiropractic students and trainees pursuant to Art. 2 (outside scope) of the Swiss Federal Act on Research involving Human Beings (Human Research Act, HRA). All participants provided voluntary electronic informed consent and it was communicated that participation in this study would not affect the students’ grading in any way. All methods followed relevant guidelines and regulations.

### Exposure / intervention – a new and improved chiropractic journal club

Development and implementation of the new format was led collaboratively by chiropractic clinician-scientists, clinicians, and students at Balgrist University Hospital and the University of Zurich. As a first step, we formed a new journal club committee, consisting of the head of clinical research within the chiropractic department (CAH), two PhD candidates with chiropractic clinical experience (RL, LN), a chiropractor and masters of medical education candidate (MH), and a chiropractic postgraduate resident (LH). MH, LH, and LN had first-hand experience of the previous journal club format as former students in the chiropractic medicine program.

We implemented the new chiropractic journal club format in February 2021 to bring together chiropractic students, clinicians, and researchers to critically appraise and discuss diverse and relevant clinical research topics. The chiropractic journal club is mandatory for all 6th year students within the chiropractic polyclinic rotation and postgraduate residents. To increase earlier engagement of students, the 5th year students were also invited to join, if interested and able. One month prior to the first session, an electronic needs assessment survey was conducted where potential participants were asked about their various research and clinical interests. Over one study semester (i.e., 6 months), five journal club sessions took place. All journal club sessions took place online via the ZOOM web application due to the ongoing COVID-19 pandemic public health restrictions. During the first session, participants were given a presentation about the definition and goals of EBP, and how to critically appraise a study based on an example using a risk of bias (RoB) tool [16]. Furthermore, the learning objectives of the journal club were specified.

For the following four sessions, we used ‘community of practice’ and ‘team-based learning’ as conceptual frameworks for the journal club sessions [11]. Undergraduate participants were divided into groups of two or three students and were paired up with one postgraduate resident who supported the group. Each group was asked to choose one journal club date and a study from a distributed study list that was curated by CAH, RL, and MH (see Additional file 2), related to the following study topics/designs: therapeutic/intervention studies, diagnostic studies, imaging studies, clinical practice guidelines, prognostic studies, or systematic reviews of chiropractic care or musculoskeletal disorders. The topics and study list were chosen based on the results of the initial needs assessment. Nonetheless, the students were still given the option of proposing another study of their own interest, after consultation and approval by the journal club leadership team (CAH, RL, MH). To aid with critical appraisal, participants were instructed to use a relevant risk of bias assessment tool based on study design, from the following options: the Scottish Intercollegiate Guidelines Network [16], the Joanna Briggs Institute [17], the RoB 2 tool (for assessing risk of bias in randomised trials) [18], or the AGREE II tool (appraisal of guidelines for research & evaluation II) [19]. A guidance document was created and distributed to students on tips for leading a successful chiropractic journal club discussion (see Additional file 3).

### Outcomes – Kirkpatrick’s model

Kirkpatrick’s model assesses the effectiveness of training programs at four levels: (1) reaction of the participant to the training program, (2) learning of professional knowledge or skills, (3) change of behaviour or performance, and (4) results on an organizational level [13].

Level 1 (reaction) was measured after the last journal club using two questions — “Which aspects of the chiropractic journal club were helpful?” “Which aspects of the chiropractic journal club do you think need improvement?” — requesting participant feedback to qualitatively gauge feasibility, acceptability, and areas for improvement of the journal club. Participants had the opportunity to raise three points that were helpful and three points that could be improved.

Levels 2 (learning) and 3 (behaviour) were assessed using an EBP questionnaire that has been previously validated in various healthcare teaching and learning contexts in different health professional students [20–25]. The EBP questionnaire consisted of four subscales: knowledge of EBP (EBP-K, 5 items scored on a 6-point Likert scale; score range = 5–30), attitudes toward EBP (EBP-A, 6 items scored on a 6-point Likert scale; score range = 6–36; reverse scored for analyses), personal application and use of EBP (EBP-P, 6 items scored on a 5-point Likert scale; score range = 6–30), and future use of EBP (EBP-F, 9 items scored on a 6-point Likert scale; score range = 9–54). The total score was the sum of these four subscale scores (26-items; score range = 26–150). The questions were slightly modified and adapted to our context and setting (see Additional file 4) and permission to use was obtained from the original author. The EBP questionnaire was distributed to participants via email one week before the first journal club session (12.02.2021), and again together with the short satisfaction assessment one week after the last journal club session (14.07.2021).

Level 4 (organisational level results) was not evaluated in this preliminary feasibility study. Immediately after each session participation was recorded through a short questionnaire and confirmed with the participant list extracted from the ZOOM video conferencing application. All data were captured via an automatically secured online data collection system (REDCap®, Research Electronic Data Capture) using electronic questionnaires.

### Analysis

Descriptive statistics were used to describe participants’ characteristics. We summarised continuous data using means and standard deviations, or medians and interquartile ranges (IQR), as appropriate. Total scores and EBP subscale scores (EBP-K, EBP-A, EBP-P, EBP-F) of the EBP questionnaire were indicated as raw scores and calculated as percentages and presented as medians and IQRs. This was performed to provide a better understanding of the differences between the four subscales in our study population before and after the implementation of the new journal club format.

A prespecified exploratory subgroup analysis was conducted based on actual participant journal club attendance during the semester (Group A: 3–5 sessions attended; Group B: ≤ 2 sessions attended). Additionally, we reported item-, subscale-, and full scale-level findings of the EBP questionnaire in our study population, including psychometric properties [26, 27] such as internal consistency reliability scores using Cronbach’s alpha [28] and floor and ceiling effects. Floor and ceiling effects were considered present if 15% or more of participants achieved the lowest or highest possible score [26].

Responses to Level 1 (reaction, feasibility, and feedback) questions were grouped and presented thematically. Answers that were provided in German were translated into English for thematic analysis by native German and English speakers. Data were extracted from REDCap into R (R Foundation for Statistical Computing, version 4.2.2) for processing and analysis [29].

## Results

Between 4 and 2021 and 12 February 2021, 32 students and trainees (n = 26 and n = 6, respectively) were identified as eligible for our healthcare education feasibility study and were invited to participate. 25 (78%) students completed the ‘before’ survey, and 29 (91%) the ‘after’ survey. Most of the study participants (79%) were female, with a mean age of 26 ± 4 years. 35% (n = 10) were in the 5th year of the chiropractic study program, 45% (n = 13) in 6th year, and 21% (n = 6) were postgraduate trainees. Group A (3–5 sessions attended) consisted of 12 participants, and 17 participants made up group B (≤ 2 sessions attended). Table 1 presents characteristics of the study population and subgroups.

**Table 1:** Characteristics of the full study population and groups A and B*

Characteristic	Full study population (n = 29)		Group A (n = 12)		Group B (n = 17)	
	N	%	N	%	N	%
Gender						
Female	23	79.3	10	83.3	13	76.5
Male	6	20.7	2	16.7	4	23.5
Age – mean ± SD (years)	26.3 ± 3.7		26.8 ± 2.4		25.9 ± 4.5	
Age groups (years)						
20–24	9	31.0	1	8.3	8	47.1
25–29	17	58.6	10	83.3	7	41.2
≥ 30	3	10.3	1	8.3	2	11.8
Level of training						
5th year	10	34.5	0	0	10	58.8
6th year	13	44.8	7	58.3	6	35.3
Postgraduate	6	20.7	5	41.7	1	5.9
Highest level of education						
High school	26	89.7	11	91.7	15	88.2
Bachelor	2	6.9	1	8.3	1	5.9
Doctorate	1	3.4	0	0	1	5.9

* Group A operationalised as participants that attended 3–5 journal club sessions during the semester; Group B as participants that attended ≤ 2 sessions during the semester

### EBP knowledge, attitudes, and behaviours (Kirkpatrick levels 2 and 3)

Our findings were compatible with no difference in before- and after- overall EBP scores following journal club implementation (median EBP overall percentage score before: 72.6 [IQR, 63.7–77.4], median EBP overall score after: 73.4 [IQR, 61.3–78.2]). Small before-and-after within group differences were found for the EBP subscale scores of knowledge, attitudes, personal application, and future use (Table 2). Group A participants (members who attended ≥ 3 sessions) showed a small within-group increase in EBP-K scores (80 vs. 84) following the new journal club implementation. In contrast, Group B participants (members who attended 2 or fewer sessions) showed no difference in EBP-K scores (80 vs. 80). Group A showed no within-group differences for EBP-A scores (80 vs. 78.3) and reduced EBP-P scores (54.2 vs. 41.7). This same trend was not found in Group B participants, who showed increased EBP-A scores (80 vs. 83.3) and increased EBP-P scores (41.7 vs. 45.8), respectively. Regarding the future use of EBP principles, Group A showed higher EBP-F within-group scores following the journal club (68.9 vs. 74.4) compared with Group B (72.2 vs. 71.1).

**Table 2:** EBP total and subscale median (IQR) raw scores and percentage scores*

Group and score type	Time point	Total score	EBP-K subscale score	EBP-A subscale score	EBP-P subscale score	EBP-F subscale score
Full study population –raw score	before	116 (105–122)	25 (24–27)	30 (29–32)	16 (13–20)	41 (37–46)
	after	117 (102–123)	25 (24–28)	30 (28–32)	16 (13–19)	41 (36–46)
Full study population –perc. score	before	72.6 (63.7–77.4)	80 (76–88)	80 (76.7–86.7)	41.7 (29.2–58.3)	71.1(62.2–82.2)
	after	73.4 (61.3–78.2)	80 (76–92)	80 (73.3–86.7)	41.7(29.2–54.2)	71.1(60-82.2)
Group A – raw score	before	112 (104.5–126)	25 (25-26.5)	30 (27.5–31.5)	19 (13–21)	40 (36.5–47)
	after	113 (102-128.2)	26 (24–28)	30 (28–31)	16 (14.5–23)	43 (34.5–49)
Group A – perc. score	before	69.4 (63.3–80.7)	80 (80–86)	80 (71.7–85)	54.2 (29.2–62.5)	68.9 (61.1–84.4)
	after	70.2 (61.3–82.5)	84 (76–92)	78.3 (73.3–83.3)	41.7 (36.5–70.8)	74.4 (56.7–88.9)
Group B – raw score	before	116.5 (108.2-120.2)	25 (24-28.25)	30 (30-31.75)	16 (12.5–18.5)	41.5 (37.75–44.75)
	after	118 (102–122)	25 (24–26)	31 (29–32)	17 (12–18)	41 (38–45)
Group B – perc. score	before	73 (66.3–76)	80 (76–93)	80 (80-85.8)	41.7 (27.1–52.1)	72.2 (63.9–79.4)
	after	74.2 (61.3 to 77.4)	80 (76–84)	83.3 (76.7–86.7)	45.8 (25–50)	71.1 (64.4–80)

Abbreviations: EBP-K, knowledge of EBP; EBP-A, attitudes toward EBP; EBP-P, personal application and use of EBP; EBP-F, future use of EBP; perc., percentage

* Group A = participants that attended 3–5 journal club sessions; Group B = participants that attended ≤ 2 sessions;

Full study population (N = 29), Group A (N = 12), Group B (N = 17)

**Fig. 1:**
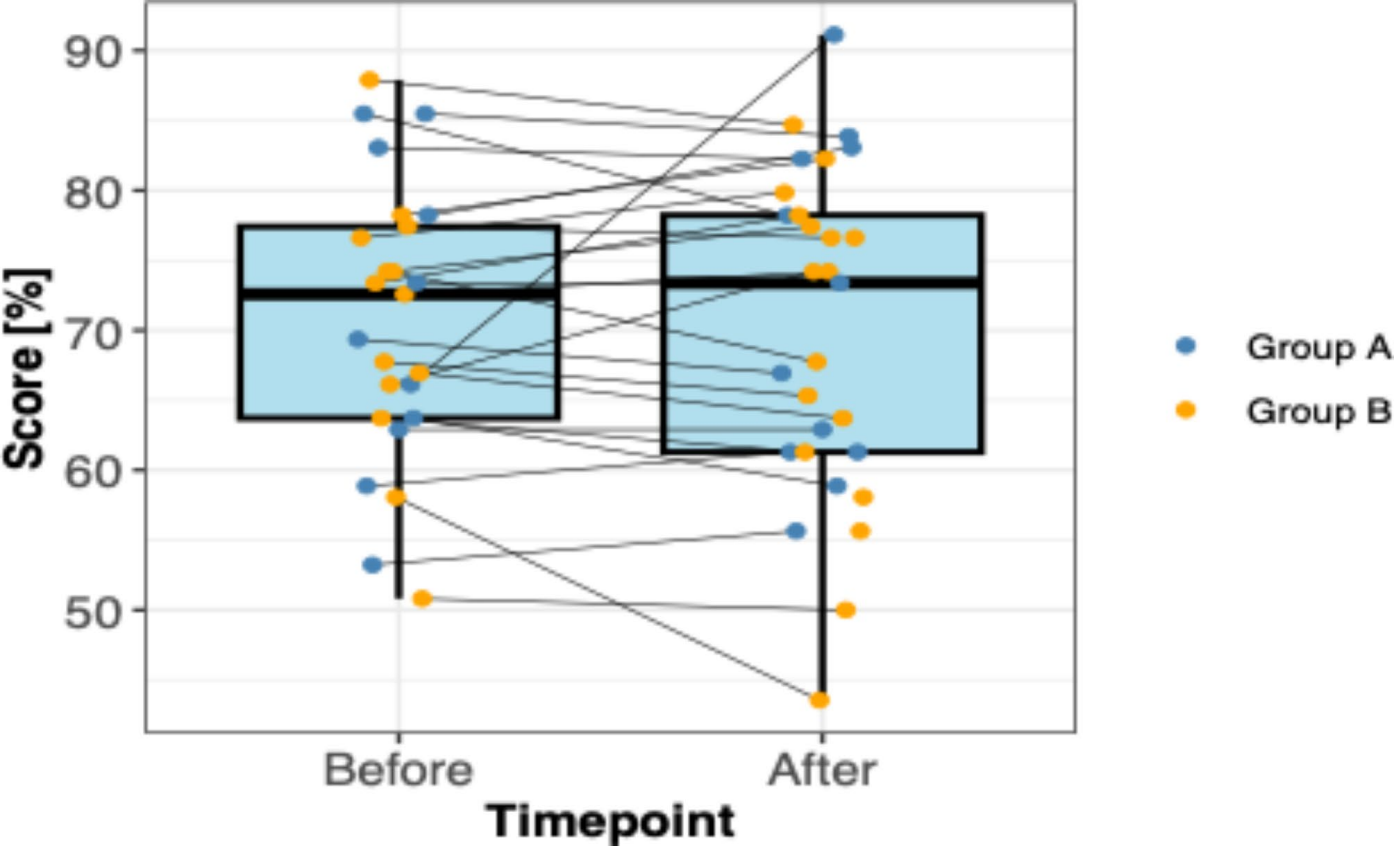
EBP total percentage scores before (n = 25) and after (n = 29) a new clinical journal club implementation during one semester among chiropractic students and trainees

Figure 1 graphically presents EBP total scores, with both full study population aggregated summaries and individual data points (stratified by subgroup) for before and after endpoints. We found considerable variation in within-person EBP total scores in both group A and group B participants.

A summary of the item-, subscale-, and full scale-level properties of the modified Johnston EBP questionnaire in our study population is presented in Additional file 5. The before mean and median total raw scores were 113.7 (12.3) and 116.0 (105.0-122.0) and the after mean and median total raw scores were 112.7 (14.6) and 117.0 (102.0-123.0). At the item-level, ceiling effects were noted in the majority of items within the subscales of “knowledge”, “attitudes” and “future use”. Floor effects were noted in the subscale category of “application”. This was seen both before and after journal club implementation. No obvious floor or ceiling effects were observed in all four subscales either before or after journal club implementation.

### Participant reaction, feedback, and satisfaction (Kirkpatrick level 1)

The themes that emerged from the data analysis of the open-ended questions allowed us to examine participant reaction and satisfaction with the new journal club. The three most common “helpful” themes (Which aspects of the chiropractic journal club were helpful?), with example representative participant responses, were:

1. Working in a team: e.g., “Grouping with a more advanced fellow”; “Discussion with a fellow”.
2. Using risk of bias assessment tools: e.g., “Working with appraisal tools”; “Working with paper-checklists”.
3. Discussing clinically relevant topics: e.g., “Talking about practice”; “Interactive discussion”.

The three most common “room for improvement” themes (Which aspects of the chiropractic journal club do you think need improvement?) were:

1. More time for discussion: e.g., “More time to discuss”; “Lack of time”.
2. Introduce risk of bias tools earlier in the program of chiropractic studies: e.g., “In 5th year already using quality assessment tools”.
3. Preference for in-person journal club rather than online: e.g., “It should be in person not online”; “Active discussion was hard online”.

## Discussion

Our preliminary before-and-after study provides an initial understanding of the associations between a newly implemented journal club and EBP knowledge, attitudes, and behaviours among chiropractic students and trainees over one academic semester. Overall, the new journal club format was feasible to implement and acceptable in a chiropractic educational setting and our findings were consistent with small before-and-after differences in the EBP factor scores for knowledge, attitudes, personal application, and future use. In addition, our study demonstrated the feasibility and acceptability of embedding chiropractic educational research within a newly implemented journal club.

This study achieved an acceptable participation proportion, with 78% (before) and 91% (after) of eligible students completing the surveys. Although the journal club was feasible to implement, all participating 5th year students were present at 2 or fewer journal club sessions. This poor participation of the 5th year students is likely the result of scheduling conflicts and the optional nature of the journal club for this group students. In contrast, 6th year students within the chiropractic department, for whom the journal club was mandatory attended 3–5 times (Group B). Our more novel journal club methods, such as the involvement of students in the design and study selection phase, were meant to promote participation and engagement. However, these methods alone may not be enough to entice student participation. Educational institutions which offer journal clubs may benefit in incentivising students for participation and providing time necessary for attendance.

Our study found an increase in the EBP domains of knowledge and future use over the course of a semester for participants who attended 3 to 5 sessions (group A) when compared to participants who attended 2 or fewer journal club sessions (group B). A similar increase in the EBP knowledge score was found in the original validation study of the Johnston questionnaire [20]. This initial validation study included a pre-post analysis of second year medical students who completed the developed questionnaire before and after a 6-module course on EBP over one academic year. Following the EBP course, a significant increase in the EBP knowledge was found (mean score pre- vs. post assessment: 4.6 vs. 4.8; p-value 0.001) with a medium effect size of 0.33 [20].

The subscore of EBP attitudes in our full study sample remained unchanged after the completion of the chiropractic journal club. These findings are similar to Cheng and colleagues [23], who compared a weekly EBP structured case conference to a didactic lecture about EBP in 94 medical students using a randomized controlled trial design. In their study, participants who were provided structured case conferences over two weeks improved in EBP knowledge and EBP practice. In contrast, our study did not find an increase in the EBP subscore of personal application after journal club participation. This finding may be due to the timing at which our last study survey was administered to participants. In late July, Swiss chiropractic students are in preparation for both curriculum-based and federal examinations; giving them less time to engage with both patients and the medical literature, and potentially limiting thoughts of personal application.

In an international sample of clinicians, Shi et al. showed that the knowledge subscore can trend towards a celling effect (all 5 knowledge sub score items demonstrated this effect) in a diverse group of physicians, nurses, occupational therapists, physical therapists, and psychologists. We found similar results in this sample of chiropractic students, with 4 out of 5 knowledge subscale items demonstrating a ceiling effect. As discussed by Shi et al., the ceiling effects may be a result of the high priority clinical education settings now place on EBP teaching [24]. In addition, our sample of chiropractic students also showed ceiling effects in 5 of 6 attitude subscale questions and 7 of 9 future use subscale questions which may demonstrate high affinity towards EBP constructs even prior to the journal club. However, a published article on self-reported attitudes, skills and use of evidence-based practice among Swiss chiropractors showed that 52% of respondents did not understand the full definition of EBP, which recognises that a patient’s preference also has to be taken into account for EBP [30]. Other national surveys of postgraduate chiropractors from the Canada, Sweden and the U.S., showed low to moderate subscores in EBP knowledge and use [31–33]. More practice-based studies are needed to better understand how views towards EBP change when students transition into clinical practice settings.

Overall, the small differences observed between the before and after measurements generates some testable hypotheses. First, participants’ attendance (or lack thereof) in journal club sessions may have played a role. Notably, participants in Group A (more attendance) showed higher scores in both the knowledge and future use subscales, whereas Group B (less attendance) had no change in these two subscales. Second, we acknowledge that explicit teaching about EBP concepts and values occurred only during the first journal club session. It is plausible that journal club sessions that integrate critical appraisal of research reports with more content on EBP concepts, behaviours, and values could potentially yield more meaningful improvement in the knowledge, attitudes, personal application, and future use of EBP.

Our course evaluation according to Kirkpatrick’s model showed that the team-based learning approach, introduced risk of bias assessment tools, and interactive clinically relevant discussions were highly valued by the students and trainees. The most frequently mentioned point for improvement was that the course should be held on-site and in-person rather than online. The journal club was initially planned to be fully in-person sessions throughout the Spring semester, but later changed to an online format due to rapidly changing COVID-19 public health guidance. This feedback was implemented the following semester, as the chiropractic journal club moved to a hybrid format.

In summary, an undergraduate student-led, team-based journal club format was shown to be feasible and highly valued by chiropractic students. Based on student feedback, the journal club will be repeated with slight modifications, but always with the long-term goal to promote EBP and inspire lifelong learning in chiropractic students. To improve behaviours of EBP personal application, future journal club sessions may select studies not only through student interest, but also in the context of common patient cases seen at Balgrist University Hospital. It may also be prudent for follow-up EBP questionnaires to be provided to participants earlier and not coinciding with student examinations.

### Strengths and limitations

A strength of our study is the methodology’s grounding in established conceptual frameworks, including ‘community of practice’ and ‘team-based learning’ for the journal club intervention and ‘Kirkpatrick’s model’ for the measurement of EBP. This study is one of only a few investigating EBP in chiropractic education and helps to fill an important knowledge gap.

Our study has important limitations. We acknowledge that our study is a small, exploratory feasibility study that used an uncontrolled before-and-after design, which limits inferences that can be made about the effects of the new journal club. It is therefore uncertain whether our findings are a result of the journal club intervention, regression to the mean, or other factors that may have influenced EBP knowledge, attitudes, and behaviours over the course of the study semester. Our results on journal club implementation and EBP outcomes should be considered preliminary evidence and interpreted cautiously.

### Future recommendations

Given the paucity of research on the use of journal clubs in chiropractic education, future studies should focus on developing larger student cohorts with lengthier follow-up periods to better assess outcomes related to EBP. In addition, it would be important to consider outcomes at Level 4 of Kirkpatrick’s model. This level would assess the impact of the new journal club as a healthcare education improvement intervention at the level of the organization. However, the ability to determine which specific outcomes and organizational impact occurred due to student participation in a new journal club format make measuring results at the organizational level quite challenging. Conceivable metrics on the educational program level might be, for example, the proportion of students that get involved in research projects, the proportion of students that choose to write a nonmandatory clinical research dissertation (i.e., known as a “Doktorarbeit” within the Swiss medical education system), or student performance in EBP-related topics of the final federal exam for full licensure in Switzerland. By including Level 4 evaluation in a future study, it would be possible to provide a more comprehensive understanding of the impact and effects of the assessed healthcare education improvement intervention. Furthermore, there is a need for randomized controlled trials in the larger medical education space to assess the effectiveness of journal clubs on the beliefs towards EBP in attending participants.

## Conclusion

Our study suggests that embedding chiropractic educational research within a newly implemented journal club is feasible and acceptable. Small before-and-after differences in the EBP factor scores for knowledge, attitudes, personal application, and future use were observed in chiropractic students and postgraduate trainees. Causal inferences about the effect of the new journal club on EBP knowledge, attitudes, personal application, and future use should be avoided.

### Electronic supplementary material

Below is the link to the electronic supplementary material.

**Additional file 1:** SQUIRE-EDU checklist; **Additional file 2:** Information about each journal club session; **Additional file 3:** The Chiro Journal Club (Chiro JC): 7 simple steps to success; **Additional file 4:** Modified Johnston EBP questionnaire; **Additional file 5:** Item-level and subscale score analysis

## Data Availability

The datasets used and/or analysed during the current study are available from the corresponding author on reasonable request.

## References

[1] Linzer M (1987). The journal club and medical education: over one hundred years of unrecorded history. Postgrad Med J.

[2] Valentini RP, Daniels SR (1997). The journal club. Postgrad Med J.

[3] Sackett D, Straus S, Richardson W, Rosenberg W, Haynes R (2000). Evidence-based medicine: how to practice and teach EBM.

[4] Sagheb MM, Amini M, Saber M, Moghadami M, Nabiei P, Khalili R (2018). Teaching evidence-based medicine (EBM) to undergraduate medical students through flipped classroom approach. Shiraz E-Med J.

[5] Dinkevich E, Markinson A, Ahsan S, Lawrence B (2006). Effect of a brief intervention on evidence-based medicine skills of pediatric residents. BMC Med Educ.

[6] Dyke P, Jamrozik K, Plant AJ (2001). A randomized trial of a problem-based learning approach for teaching epidemiology. Acad Med.

[7] Leung GM, Johnston JM (2006). Evidence-based medical education - *quo vadis* ?: evidence-based medical education. J Eval Clin Pract.

[8] Green BN, Johnson CD (2007). Use of a modified journal club and letters to editors to teach critical appraisal skills. J Allied Health.

[9] Dirschl DR, Tornetta P, Bhandari M (2003). Designing, conducting, and evaluating journal clubs in orthopaedic surgery. Clin Orthop.

[10] Hartzell JD, Veerappan GR, Posley K, Shumway NM, Durning SJ (2009). Resident run journal club: a model based on the adult learning theory. Med Teach.

[11] Lave JWE (1991). Situated learning: legitimate peripheral participation (learning in doing: social, cognitive and computational perspectives).

[12] Deenadayalan Y, Grimmer-Somers K, Prior M, Kumar S (2008). How to run an effective journal club: a systematic review: how to run an effective journal club. J Eval Clin Pract.

[13] Kirkpatrick D (1967). Evaluation of training. New York.

[14] Hofstetter L, Häusler M, Mühlemann M, Nyirö L, Mühlemann D, Hincapié CA (2022). Musculoskeletal healthcare at a swiss university hospital chiropractic medicine outpatient clinic in 2019: a health services research study. Chiropr Man Ther.

[15] Ogrinc G, Armstrong GE, Dolansky MA, Singh MK, Davies L (2019). SQUIRE-EDU (standards for quality improvement reporting excellence in education): publication guidelines for educational improvement. Acad Med.

[16] Scottish Intercollegiate Guidelines Network (SIGN). SIGN Checklists for risk of bias assessment. Available at: https://testing36.scot.nhs.uk. Accessed 11 Oct 2021.

[17] Joanna Briggs Institute. Critical appraisal tools. Available at: https://jbi.global/critical-appraisal-tools. Accessed 11 Oct 2021.

[18] Sterne JAC, Savović J, Page MJ, Elbers RG, Blencowe NS, Boutron I (2019). RoB 2: a revised tool for assessing risk of bias in randomised trials. BMJ.

[19] Brouwers MC, Kho ME, Browman GP, Burgers JS, Cluzeau F, Feder G (2010). AGREE II: advancing guideline development, reporting and evaluation in health care. J Clin Epidemiol.

[20] Johnston JM, Leung GM, Fielding R, Tin KYK, Ho L-M (2003). The development and validation of a knowledge, attitude and behaviour questionnaire to assess undergraduate evidence-based practice teaching and learning. Med Educ.

[21] Leung GM, Johnston JM, Tin KYK, Wong IOL, Ho L-M, Lam WWT (2003). Randomised controlled trial of clinical decision support tools to improve learning of evidence based medicine in medical students. BMJ.

[22] Johnston JM, Schooling CM, Leung GM (2009). A randomised-controlled trial of two educational modes for undergraduate evidence-based medicine learning in Asia. BMC Med Educ.

[23] Cheng HM, Guo FR, Hsu TF, Chuang SY, Yen HT, Lee FY (2012). Two strategies to intensify evidence-based medicine education of undergraduate students: a randomised controlled trial. Ann Acad Med Singap.

[24] Shi Q, Chesworth BM, Law M, Haynes RB, MacDermid JC (2014). A modified evidence-based practice knowledge, attitudes, behaviour and decisions/outcomes questionnaire is valid across multiple professions involved in pain management. BMC Med Educ.

[25] Arumugam V, MacDermid JC, Walton D, Grewal R (2018). Attitudes, knowledge and behaviors related to evidence-based practice in health professionals involved in pain management. Int J Evid Based Healthc.

[26] Terwee CB, Bot SDM, de Boer MR, van der Windt DAWM, Knol DL, Dekker J (2007). Quality criteria were proposed for measurement properties of health status questionnaires. J Clin Epidemiol.

[27] Mokkink LB, Terwee CB, Patrick DL, Alonso J, Stratford PW, Knol DL (2010). The COSMIN study reached international consensus on taxonomy, terminology, and definitions of measurement properties for health-related patient-reported outcomes. J Clin Epidemiol.

[28] Tavakol M, Dennick R (2011). Making sense of Cronbach’s alpha. Int J Med Educ.

[29] R Core Team (2018). R: a language and environment for statistical computing.

[30] Albisser A, Schweinhardt P, Bussières A, Baechler M (2022). Self-reported attitudes, skills and use of evidence-based practice among swiss chiropractors: a national survey. Chiropr Man Ther.

[31] Leach MJ, Palmgren PJ, Thomson OP, Fryer G, Eklund A, Lilje S (2021). Skills, attitudes and uptake of evidence-based practice: a cross-sectional study of chiropractors in the swedish Chiropractic Association. Chiropr Man Ther.

[32] Bussières AE, Terhorst L, Leach M, Stuber K, Evans R, Schneider MJ. Self-reported attitudes, skills and use of evidence-based practice among canadian doctors of chiropractic: a national survey. J Can Chiropr Assoc. 2015 Dec;59(4):332–48. PMC4711333 26816412

[33] Schneider MJ, Evans R, Haas M, Leach M, Hawk C, Long C (2015). US chiropractors’ attitudes, skills and use of evidence-based practice: a cross-sectional national survey. Chiropr Man Ther.

