# Correction: Feasibility of a new clinical journal club implementation and its association with knowledge, attitudes, and application of evidence-based practice among chiropractic students and trainees: a before-and-after healthcare education improvement study

**DOI:** 10.1186/s12998-023-00508-x

**Published:** 2023-09-26

**Authors:** Melanie Häusler, Rahim Lalji, Léonie Hofstetter, Cesar A. Hincapié

**Affiliations:** 1 EBPI-UWZH Musculoskeletal Epidemiology Research Group, University of Zurich and Balgrist University Hospital, Forchstrasse 340, Zurich, 8008 Switzerland; 2 Department of Chiropractic Medicine, Faculty of Medicine, Balgrist University Hospital, University of Zurich, Zurich, Switzerland; 3 Epidemiology, Biostatistics and Prevention Institute (EBPI), University of Zurich, Zurich, Switzerland; 4 Balgrist University Hospital, University Spine Centre Zurich (UWZH), University of Zurich, Zurich, Switzerland

Chiropractic & Manual Therapies (2023) 31:22

10.1186/s12998-023-00494-0.

After publication of this article [1], the authors reported that in the Methods-section of the abstract, “between 1 and 2021 and 31 July 2021” should have read “between 1 February 2021 and 31 July 2021”.

The original article [1] has been corrected.
