# Supplementary material for: Feasibility of a new clinical journal club implementation and its association with knowledge, attitudes, and application of evidence-based practice among chiropractic students and trainees: a before-and-after healthcare education improvement study
Source: Chiropr Man Therap. 2023 Jul 24;31:22. doi: 10.1186/s12998-023-00494-0 (PMC10367234; doi:10.1186/s12998-023-00494-0)
Supplement: Supplementary file 1 — Additional file 1: SQUIRE-EDU checklist; Additional file 2: Information about each journal club session; Additional file 3: The Chiro Journal Club (Chiro JC): 7 simple steps to success; Additional file 4: Modified Johnston EBP questionnaire; Additional file 5: Item-level and subscale score analysis [file 12998_2023_494_MOESM1_ESM.docx]

**Title:** Feasibility of a new clinical journal club implementation and its association with knowledge, attitudes, and application of evidence-based practice among chiropractic students and trainees: a before-and-after healthcare education improvement study

**Authors:** Melanie Häusler DCM,^1,2,*^ Rahim Lalji DC MSc,^1,2,3,4^ Léonie Hofstetter DCM,^1,2^ Cesar A. Hincapié DC PhD^1,2,3,4^

**Affiliations:**

^1^ EBPI-UWZH Musculoskeletal Epidemiology Research Group, University of Zurich and Balgrist University Hospital, Zurich, Switzerland

^2^ Department of Chiropractic Medicine, Faculty of Medicine, Balgrist University Hospital and University of Zurich, Zurich, Switzerland

^3^ Epidemiology, Biostatistics and Prevention Institute (EBPI), University of Zurich, Zurich, Switzerland

^4^ University Spine Centre Zurich (UWZH), Balgrist University Hospital, University of Zurich, Zurich, Switzerland

**Table of Contents**

Additional File 1: SQUIRE-EDU checklist 2

Additional File 2: Information about each journal club session 7

Additional File 3: The Chiro Journal Club (Chiro JC): 7 simple steps to success 8

Additional File 4: Study EBP questionnaire – adapted from original Johnston EBP questionnaire) 9

Additional File 5: Item-level and subscale score analysis 11

## Additional File 1: SQUIRE-EDU checklist

| **Squire-EDU item** | **Example of text in manuscript** |
| --- | --- |
| **Title and abstract** | |
| **EDU 1**. Indicate that the manuscript concerns efforts to improve health professions education systems and learning | Pg 1, Ln 1:  “Feasibility of a new clinical journal club implementation and its association with knowledge, attitudes, and application of evidence-based practice among chiropractic students and trainees: a before-and-after healthcare education improvement study”  Pg 4, Ln 102:  “The objectives of this study were to (1) assess the feasibility of the new journal club implementation, and (2) estimate associations between the new journal club implementation and EBP characteristics (ie, knowledge, attitudes, and application of EBP) among chiropractic medicine students and trainees over one academic semester.” |
| **EDU 2.** Keywords include a focus on education and learning | Pg 3, Ln 66:  “Medical education”, “Health personnel education” |
| **Introduction: Why did you start?** | |
| **EDU 3.** Description of the nature and significance of the need for change in the local educational system | Pg 4, Ln 88:  “While it was routine for experienced researchers to lead discussions and critically appraise the literature, chiropractic students with clinical interests and less research experience often found this format challenging.” |
| **EDU 4**. Available knowledge Summary of what is currently known about the problem, including relevant previous studies | Pg 4, Ln 94:  “Similar experiences have been reported in other academic health institutions, with reports of medical journal club content lacking clinical relevance and containing an unnecessarily large focus on biostatistics.” |
| **EDU 5.** Identify the guiding theory (learning, change, implementation, or other) and how it aligns with the need for change in the local educational system | Pg 4, Ln 96:  “Given this experience, a new journal club format was created based on the conceptual frameworks of ‘community of practice’ and ‘team-based learning’, and following established recommendations on how to run an effective journal club |
| **EDU 6.** Purpose of the project and of this report | Pg 4, Ln 102:  “The objectives of this study were to (1) assess the feasibility of the new journal club implementation, and (2) estimate associations between the new journal club implementation and EBP characteristics (ie, knowledge, attitudes, and application of EBP) among chiropractic medicine students and trainees over one academic semester” |
| **Methods: What did you do?** | |
| **EDU 7a.** Contextual elements for learning (e.g., setting, program, people, resources, social, geopolitical influences) before the intervention(s) | Pg 4, Ln 114:  “During the last year, students spend 6 months in the outpatient chiropractic polyclinic [14] and 6 months rotating through other specialties (e.g., orthopaedics, rheumatology, neurology, radiology, and sports medicine).” |
| **EDU 7b**. The interrelationships between the contextual elements and the local educational and healthcare systems before the intervention(s) | Pg 6, Ln 146:  “The chiropractic journal club is mandatory for all 6th year students within the chiropractic polyclinic rotation and postgraduate residents. To increase earlier engagement of students, the 5th year students were also invited to join, if interested and able.” |
| **EDU 8a.** Description of the primary interventions and co-interventions (e.g., faculty or tool development) | Pg 6, Ln 144:  “We implemented the new chiropractic journal club format in February 2021 to bring together chiropractic students, clinicians, and researchers to critically appraise and discuss diverse and relevant clinical research topics.” |
| **EDU 8b.** Specify how the interprofessional education team (e.g., faculty, staff, patients, and learners) was part of the design of the intervention | Pg 5, Ln 138:  “As a first step, we formed a new journal club committee, consisting of the head of clinical research within the chiropractic department (CAH), two PhD candidates with chiropractic clinical experience (RL, LN), a chiropractor and masters of medical education candidate (MH), and a chiropractic postgraduate resident (LH).” |
| **EDU 9a.** Approach used to understand the impact of the educational intervention(s) on the learner and beyond, such as impact on patients, families, the community, faculty, educational program, or the healthcare system | Pg 7, Ln 176:  “Kirkpatrick's model assesses the effectiveness of training programs at four levels: 1) reaction of the participant to the training program, 2) learning of professional knowledge or skills, 3) change of behaviour or performance, and 4) results on an organizational level.” |
| **EDU 9b.** Approach to assess the fidelity of and the iterative changes to the planned intervention(s) over time | Pg 7, Ln 194:  “The EBP questionnaire was distributed to participants via email one week before the first journal club session (12.02.2021), and again together with the short satisfaction assessment one week after the last journal club session (14.07.2021).”  Pg 8, Ln 199:  “Immediately after each session participation was recorded through a short questionnaire and confirmed with the participant list extracted from the ZOOM video conferencing application.” |
| **EDU 10.** Quantitative and/or qualitative measures chosen to assess the educational processes and outcomes on learners, faculty, educational programs, patients, families, healthcare systems, or communities | Pg 7, Ln 176:  “Kirkpatrick's model assesses the effectiveness of training programs at four levels: 1) reaction of the participant to the training program, 2) learning of professional knowledge or skills, 3) change of behavior or performance, and 4) results on an organizational level” |
| **EDU 11a.** Qualitative and quantitative methods used to draw inferences from the data | Pg 8, Ln 213:  “A prespecified exploratory subgroup analysis was conducted based on actual participant journal club attendance during the semester (Group A: 3-5 sessions attended; Group B: ≤ 2 sessions attended).”  Pg 8, Ln 208:  “Total scores and EBP subscale scores (EBP-K, EBP-A, EBP-P, EBP-F) of the EBP questionnaire were indicated as raw scores and calculated as percentages and presented as medians and IQRs.” |
| **EDU 11b.** Methods for understanding variation within the data, including the effects of time as a variable | Pg 8, Ln 215:  “Additionally, we reported item-, subscale-, and full scale-level findings of the EBP questionnaire in our study population, including psychometric properties such as internal consistency reliability scores using Cronbach’s alpha.” |
| **EDU 12.** Approaches to address vulnerability of learner participants | Pg 5, Ln 127:  “The local independent research ethics committee of Canton Zurich deemed that ethical approval was not required for this healthcare education feasibility study of Swiss chiropractic students and trainees pursuant to Art. 2 (outside scope) of the Swiss Federal Act on Research involving Human Beings (Human Research Act, HRA). All participants provided voluntary electronic informed consent and it was communicated that participation in this study would not affect the students’ grading in any way. All methods followed relevant guidelines and regulations.” |
| **Results: What did you find?** | |
| **EDU 13a.** For each educational intervention and co-intervention, provide details about iterative modifications based on the assessment of the learning | Not applicable. |
| **EDU 13b.** Details of the process measures and outcomes | Pg 7, Ln 175:  “Outcomes – Kirkpatrick's model…” |
| **EDU 13c.** Contextual elements that interacted with the intervention(s) | Pg 10, Ln 244:  “Group A participants (members who attended ≥3 sessions) showed a small within-group increase in EBP-K scores (80 vs. 84) following the new journal club implementation. In contrast, Group B participants (members who attended 2 or fewer sessions) showed no difference in EBP-K scores (80 vs. 80)” |
| **EDU 13d.** Observed associations between outcomes, interventions, and relevant contextual elements | Pg 10, Ln 242:  “Small before-and-after within group differences were found for the EBP subscale scores of knowledge, attitudes, personal application, and future use.”  Pg 15, Ln 328:  “Following the EBP course, a significant increase in the EBP knowledge was found (mean score pre- vs. post assessment: 4.6 vs. 4.8; p-value 0.001) with a medium effect size of 0.33.” |
| **EDU 13e.** Unintended consequences such as unexpected benefits, problems, failures, or costs associated with the intervention(s) | Pg 10, Ln 247:  “Group A showed no within-group differences for EBP-A scores (80 vs. 78.3) and reduced EBP-P scores (54.2 vs. 41.7). This same trend was not found in Group B participants, who showed increased EBP-A scores (80 vs. 83.3) and increased EBP-P scores (41.7 vs. 45.8), respectively. Regarding the future use of EBP principles, Group A showed higher EBP-F within-group scores following the journal club (68.9 vs. 74.4) compared with Group B (72.2 vs. 71.1).” |
| **EDU 13f.** Details about missing data | Pg 8, Ln 213:  “A prespecified exploratory subgroup analysis was conducted based on actual participant journal club attendance during the semester (Group A: 3-5 sessions attended; Group B: ≤ 2 sessions attended).”  Pg 9, Ln 229:  “25 (78%) students completed the ‘before’ survey, and 29 (91%) the ‘after’ survey.” |
| **Discussion: What does it mean?** | |
| **EDU 14.** Connect the findings to the guiding theory (learning, change, implementation, other) used to direct the change in the local educational system | Pg 17, Ln 358:  “Our course evaluation according to Kirkpatrick’s model showed that the team-based learning approach, introduced risk of bias assessment tools, and interactive clinically relevant discussions were highly valued by the students and trainees.” |
| **EDU 15a.** Nature of the association between the intervention(s) and the outcomes | Pg 15, Ln 305:  “Overall, the new journal club format was feasible to implement and acceptable in a chiropractic educational setting and our findings were consistent with small before-and-after differences in the EBP factor scores for knowledge, attitudes, personal application, and future use.” |
| **EDU 15b.** Comparison of results with findings from other publications | Pg 16, Ln 336:  “In contrast, our study did not find an increase in the EBP subscore of personal application after journal club participation. This finding may be due to the timing at which our last study survey was administered to participants.” |
| **EDU 15c.** Include the impact of the intervention(s) on learners, faculty, educational program, patients, families, healthcare systems, or communities | Pg 15, Ln 305:  “Overall, the new journal club format was feasible to implement and acceptable in a chiropractic educational setting and our findings were consistent with small before-and-after differences in the EBP factor scores for knowledge, attitudes, personal application, and future use.” |
| **EDU 15d.** Reasons for any differences between observed and anticipated outcomes, including the influence of context | Pg 16, Ln 337:  “This finding may be due to the timing at which our last study survey was administered to participants. In late July, Swiss chiropractic students are in preparation for both curriculum-based and federal examinations; giving them less time to engage with both patients and the medical literature, and potentially limiting thoughts of personal application.” |
| **EDU 15e.** Costs and strategic trade-offs, including opportunity costs | Not applicable. |
| **EDU 16a.** Limits to the generalizability of the work | Pg 17, Ln 381:  “We acknowledge that our study is a small, exploratory feasibility study that used an uncontrolled before-and-after design, which limits inferences that can be made about the effects of the new journal club.”  Pg 18, Ln 386:  “Our results on journal club implementation and EBP outcomes should be considered preliminary evidence and interpreted cautiously.” |
| **EDU 16b.** Factors that might have limited internal validity such as confounding, bias, or imprecision in the design, methods, measurement, or analysis | Pg 17, Ln 381:  “We acknowledge that our study is a small, exploratory feasibility study that used an uncontrolled before-and-after design, which limits inferences that can be made about the effects of the new journal club. It is therefore uncertain whether our findings are a result of the journal club intervention, regression to the mean, or other factors that may have influenced EBP knowledge, attitudes, and behaviours over the course of the study semester.” |
| **EDU 16c.** Efforts made to minimize and adjust for limitations | Pg 17, Ln 381:  “We acknowledge that our study is a small, exploratory feasibility study that used an uncontrolled before-and-after design, which limits inferences that can be made about the effects of the new journal club.”  Pg 18, Ln 386:  “Our results on journal club implementation and EBP outcomes should be considered preliminary evidence and interpreted cautiously.” |
| **EDU 17a.** Usefulness of the work | Pg 17, Ln 378:  “This study is one of only a few investigating EBP in chiropractic education and helps to fill an important knowledge gap.” |
| **EDU 17b.** Scalability of the work to other learners and contexts | Pg 17, Ln 367:  “Based on student feedback, the journal club will be repeated with slight modifications, but always with the long-term goal to promote EBP and inspire lifelong learning in chiropractic students.” |
| **EDU 17c**. Potential for spread to other contexts | Pg 17, Ln 369:  “To improve behaviours of EBP personal application, future journal club sessions may select studies not only through student interest, but also in the context of common patient cases seen at Balgrist University Hospital.” |
| **EDU 17d.** Lessons learned for clinical practice, education, and policy | Pg 17, Ln 367:  “Based on student feedback, the journal club will be repeated with slight modifications, but always with the long-term goal to promote EBP and inspire lifelong learning in chiropractic students.” |
| **EDU 17e.** Suggested next steps | Pg 18. Ln 390:  “Given the paucity of research on the use of journal clubs in chiropractic education, future studies should focus on developing larger student cohorts with lengthier follow-up periods to better assess outcomes related to EBP. Furthermore, there is a need for randomized controlled trials in the larger medical education space to assess the effectiveness of journal clubs on the beliefs towards EBP in attending participants.” |
| **Other information** | |
| **EDU 18.** Funding | Pg 23, Ln 533:  “The current study received no funding.” |

## Additional File 2: Information about each journal club session

| **Journal club session** | **Date** | **Study design/topic** | **Title of chosen journal article** | **Design** | **RoB-Tool** |
| --- | --- | --- | --- | --- | --- |
| 1 | 17.02.2021 | Therapeutic | Physical therapy referral from primary care for acute back pain with sciatica: a randomized controlled trial [1] | Randomized control trial | SIGN checklist for randomized controlled trials |
| 2 | 07.04.2021 | Prognostic | Prognostic indicators for poor outcomes in low back pain patients consulted in primary care [2] | Cohort study | SIGN checklist for cohort studies |
| 3 | 05.05.2021 | Clinical practice guideline | Non-invasive treatments for acute, subacute, and chronic low back pain: a clinical practice guideline from the American College of Physicians [3] | Clinical practice guideline | AGREE 2 |
| 4 | 02.06.2021 | Therapeutic | Comparative clinical effectiveness of nonsurgical treatment methods in patients with lumbar spinal stenosis: a randomized controlled trial [4] | Randomized control trial | RoB 2 tool |
| 5 | 14.07.2021 | Diagnostic | Systematic review of patient history and physical examination to diagnose chronic low back pain originating from the facet joints [5] | Systematic review | SIGN checklist for systematic review |
| Abbreviations: AGREE 2, Appraisal of guidelines research and evaluation; RoB 2, Risk of bias 2; SIGN, Scottish intercollegiate guidelines network | | | | | |

**References:**

1. Fritz JM, Lane E, McFadden M, Brennan G, Magel JS, Thackeray A, et al. Physical therapy referral from primary care for acute back pain with sciatica: a randomized controlled trial. Ann Intern Med. 2021;174:8–17.

2. Cruz EB, Canhão H, Fernandes R, Caeiro C, Branco JC, Rodrigues AM, et al. Prognostic indicators for poor outcomes in low back pain patients consulted in primary care. PloS One. 2020;15:e0229265.

3. Qaseem A, Wilt TJ, McLean RM, Forciea MA, Clinical Guidelines Committee of the American College of Physicians. Noninvasive treatments for acute, subacute, and chronic low back pain: a clinical practice guideline from the American College of Physicians. Ann Intern Med. 2017;166:514–30.

4. Schneider MJ, Ammendolia C, Murphy DR, Glick RM, Hile E, Tudorascu DL, et al. Comparative clinical effectiveness of nonsurgical treatment methods in patients with lumbar spinal stenosis: a randomized clinical trial. JAMA Netw Open. 2019;2:e186828.

5. Maas ET, Juch JNS, Ostelo RWJG, Groeneweg JG, Kallewaard JW, Koes BW, et al. Systematic review of patient history and physical examination to diagnose chronic low back pain originating from the facet joints. Eur J Pain. 2017;21:403–14.

## Additional File 3: The Chiro Journal Club (Chiro JC): 7 simple steps to success

**Tips for leading a successful Chiro JC discussion.**

1. **Create your Chiro JC group.**

Groups of 3 consist of 2 UnderAssistants (UAs) paired with 1 clinical fellow/academic trainee. (MM, LN, LH, MH, RL)

1. **As a group choose a date and topic to lead the Chiro JC discussion.**

Dates: 17.02.2021, 07.04.2021, 05.05.2021, 02.06.2021, 14.07.2021

Topics: Diagnosis, Imaging, Clinical Practice Guidelines, Prognosis, Systematic reviews

Send an email to RL and LN indicating which date and topic your group has chosen.

1. **As a group choose a paper and pick a risk of bias (RoB) critical appraisal tool to use.**

RoB tools are study design specific and have helpful notes: [SIGN checklists](https://www.sign.ac.uk/what-we-do/methodology/checklists/) or [JBI checklists](https://jbi.global/critical-appraisal-tools)

Send an email—as soon as decided or at least 4 weeks before your Chiro JC session—to RL and LN indicating which paper and RoB tool your group has chosen.

1. **Each person in your group individually reads the paper and performs an independent RoB assessment using your chosen RoB tool.**

For your critical appraisal summary think about:

- Top 3 strengths of the study
- Top 3 weaknesses of the study
- Top 3 clinical implications (clinical relevance/application) of the study

1. **Have a group meeting to develop your group RoB consensus critical appraisal.**

Develop group consensus on each RoB question/item from your RoB tool.
Develop group consensus on the overall quality/RoB rating of the study.
Develop group consensus on the Top 3s (strengths, weaknesses, clinical implications).

1. **On the date of your Chiro JC session, expect that everyone has read your JC paper.**

If you choose to briefly summarize the study, this should take no longer than 3 minutes.

JC sessions are not a group presentation, but rather a group led open and interactive discussion.

1. **On the date of your Chiro JC session, lead an open and active discussion of your JC paper.**

An easy way to structure the Chiro JC discussion is to guide the session through your group RoB consensus assessment. If your group chooses to use slides, they should be focused on a guided discussion of:

- - Your group’s consensus ratings for individual RoB questions/items
  - Your group’s consensus on study overall quality/RoB rating
  - Your group’s consensus on Top 3s (strengths, weaknesses, clinical implications)

## Additional File 4: Study EBP questionnaire – adapted from original Johnston EBP questionnaire [1])

| **EBP – Knowledge of EBP subscale (EBP-K)** | | | | | | | | | |
| --- | --- | --- | --- | --- | --- | --- | --- | --- | --- |
| 1. Evidence-based practice requires the use of critical appraisal skills to ensure the quality of all the research papers retrieved. | | | | | | | | | |
| 1 Strongly disagree | 2 Disagree | | 3 Somewhat disagree | | 4 Somewhat agree | | 5 Agree | | 6 Strongly agree |
| 1. Effective searching skills ⁄ easy access to bibliographic databases and evidence sources are essential to practising evidence-based practice. | | | | | | | | | |
| 1 Strongly disagree | 2 Disagree | | 3 Somewhat disagree | | 4 Somewhat agree | | 5 Agree | | 6 Strongly agree |
| 1. Critically appraised evidence should be appropriately applied to the patient using clinical judgment and experience. | | | | | | | | | |
| 1 Strongly disagree | 2 Disagree | | 3 Somewhat disagree | | 4 Somewhat agree | | 5 Agree | | 6 Strongly agree |
| 1. The evidence-based practice process requires the appropriate identification and formulation of clinical questions. | | | | | | | | | |
| 1 Strongly disagree | 2 Disagree | | 3 Somewhat disagree | | 4 Somewhat agree | | 5 Agree | | 6 Strongly agree |
| 1. Practicing evidence-based practice increases the certainty that the proposed treatment is effective. | | | | | | | | | |
| 1 Strongly disagree | 2 Disagree | | 3 Somewhat disagree | | 4 Somewhat agree | | 5 Agree | | 6 Strongly agree |
| **EBP – Attitudes toward EBP subscale (EBP-A)** [the EBP-A subscale is reverse scored for analysis] | | | | | | | | | |
| 1. If evidence-based practice is valid, then anyone can see patients and do what chiropractors do. | | | | | | | | | |
| 1 Strongly disagree | 2 Disagree | | 3 Somewhat disagree | | 4 Somewhat agree | | 5 Agree | | 6 Strongly agree |
| 1. There is no reason for me personally to adopt evidence-based practice because it is just a fad that will pass with time. | | | | | | | | | |
| 1 Strongly disagree | 2 Disagree | | 3 Somewhat disagree | | 4 Somewhat agree | | 5 Agree | | 6 Strongly agree |
| 1. Evidence-based practice is "cook-book" chiropractic that disregards clinical experience. | | | | | | | | | |
| 1 Strongly disagree | 2 Disagree | | 3 Somewhat disagree | | 4 Somewhat agree | | 5 Agree | | 6 Strongly agree |
| 1. Chiropractors, in general, should not practice evidence-based practice because chiropractic is about people and patients, not statistics. | | | | | | | | | |
| 1 Strongly disagree | 2 Disagree | | 3 Somewhat disagree | | 4 Somewhat agree | | 5 Agree | | 6 Strongly agree |
| 1. Evidence-based practice ignores the "art" of chiropractic. | | | | | | | | | |
| 1 Strongly disagree | 2 Disagree | | 3 Somewhat disagree | | 4 Somewhat agree | | 5 Agree | | 6 Strongly agree |
| 1. Previous work experience is more important than research findings in choosing the best treatment available for a patient. | | | | | | | | | |
| 1 Strongly disagree | 2 Disagree | | 3 Somewhat disagree | | 4 Somewhat agree | | 5 Agree | | 6 Strongly agree |
| **EBP – Personal application and use of EBP subscale (EBP-P)** | | | | | | | | | |
| 1. How frequently do you access chiropractic/medical evidence from a textbook? | | | | | | | | | |
| 1 Never | | 2 Rarely | | 3 Sometimes | | 4 Very often | | 5 Every day | |
| 1. How frequently do you access chiropractic/medical evidence in general? | | | | | | | | | |
| 1 Never | | 2 Rarely | | 3 Sometimes | | 4 Very often | | 5 Every day | |
| 1. How frequently do you access chiropractic/medical evidence on the Internet (excluding Medline and Cochrane Reviews)? | | | | | | | | | |
| 1 Never | | 2 Rarely | | 3 Sometimes | | 4 Very often | | 5 Every day | |
| 1. How frequently do you access chiropractic/medical evidence from original research papers? | | | | | | | | | |
| 1 Never | | 2 Rarely | | 3 Sometimes | | 4 Very often | | 5 Every day | |
| 1. How frequently do you access chiropractic/medical evidence from the Cochrane database? | | | | | | | | | |
| 1 Never | | 2 Rarely | | 3 Sometimes | | 4 Very often | | 5 Every day | |
| 1. How frequently do you access chiropractic/medical evidence from secondary sources such as clinical practice guidelines, the ACP Journal Club, the Journal of Evidence-Based Medicine, POEMs (Patient Oriented Evidence that Matters) or CATs (Critically Appraised Topics)? | | | | | | | | | |
| 1 Never | | 2 Rarely | | 3 Sometimes | | 4 Very often | | 5 Every day | |
| **EBP – Future use of EBP subscale (EBP-F)** | | | | | | | | | |
| 1. Compared to 1 year ago, how useful do you believe evidence-based practice will be in your future practice as a chiropractor? | | | | | | | | | |
| 1 Completely useless | 2 | | 3 | | 4 | | 5 | | 6 Very useful |
| 1. Compared to 1 year ago, how willing are you to practice evidence-based practice as a chiropractor in the future? | | | | | | | | | |
| 1 Very unwilling | 2 | | 3 | | 4 | | 5 | | 6 Very willing |
| 1. Do you personally appreciate the advantages of practicing evidence-based practice? | | | | | | | | | |
| 1 Not at all | 2 Low | | 3 Slightly | | 4 Moderately | | 5 Very | | 6 Extremely |
| 1. Evidence-based practice should be an integral part of the undergraduate chiropractic medical curriculum? | | | | | | | | | |
| 1 Not at all | 2 Low | | 3 Slightly | | 4 Moderately | | 5 Very | | 6 Extremely |
| 1. Compared to 1 year ago, how much do you support the principles of evidence-based practice? | | | | | | | | | |
| 1 Not at all | 2 Low | | 3 Slightly | | 4 Moderately | | 5 Very | | 6 Extremely |
| 1. Compared to 1 year ago, how much do you support lifelong learning using evidence-based practice competencies? | | | | | | | | | |
| 1 Not at all | 2 Low | | 3 Slightly | | 4 Moderately | | 5 Very | | 6 Extremely |
| 1. How much do you consider the practice of evidence-based practice a routine part of your learning? | | | | | | | | | |
| 1 Not at all | 2 Low | | 3 Slightly | | 4 Moderately | | 5 Very | | 6 Extremely |
| 1. How much has the practice of evidence-based practice changed the way you learn? | | | | | | | | | |
| 1 Not at all | 2 Low | | 3 Slightly | | 4 Moderately | | 5 Very | | 6 Extremely |
| 1. How easy has it been for you to practice evidence-based practice as a chiropractic medicine student in the last month? | | | | | | | | | |
| 1 Not at all | 2 Low | | 3 Slightly | | 4 Moderately | | 5 Very | | 6 Extremely |

**Reference**

1. Johnston JM, Leung GM, Fielding R, Tin KYK, Ho L-M. The development and validation of a knowledge, attitude and behaviour questionnaire to assess undergraduate evidence-based practice teaching and learning. Med Educ. 2003;37:992–1000.

**Adaptations made to the original EBP questionnaire:**

1. All terms “evidence-based medicine” were changed to “evidence-based practice”
2. All terms “doctor” were changed to “chiropractor”
3. All terms “medical evidence” were changed to “chiropractic/medical evidence”
4. EBP-F: 6. “evidence-based medicine techniques” was changed to “evidence-based practice competencies”
5. EBP-F: 9. “medical student” was changed to “chiropractic medicine student”

Consent to use the slightly modified questionnaire was granted by Prof. Janice Johnston.

## Additional File 5: Item-level and subscale score analysis

Item-level and subscale score analysis of the modified EBP questionnaire *before* implementation of a new journal club in chiropractic students and trainees (n=25)

| **Subscale and item** | **Item-level** | | | | **Subscale-level** | | | |
| --- | --- | --- | --- | --- | --- | --- | --- | --- |
|  | **Mean (SD)** | **Median (IQR)** | **Floor %** | **Ceiling %** | **Mean (SD)** | **Floor %** | **Ceiling %** | **Cronbach’s α** |
| **Knowledge (EBP-K)** | | | | | 25.6 (2.2) | 0 | 12 | 0.625 |
| EBP-K1 | 5.2 (0.5) | 5 (5-5) | 0 | **24** |  |  |  |  |
| EBP-K2 | 5.2 (0.9) | 5 (5-6) | 0 | **36** |  |  |  |  |
| EBP-K3 | 5.5 (0.6) | 6 (5-6) | 0 | **56** |  |  |  |  |
| EBP-K4 | 4.9 (0.7) | 5 (5-5) | 0 | 12 |  |  |  |  |
| EBP-K5 | 4.9 (0.8) | 5 (4-5) | 0 | **24** |  |  |  |  |
| **Attitudes (EBP-A)** | | | | | 30.2 (2.8) | 0 | 0 | 0.674 |
| EBP-A1 | 5.0 (0.6) | 5 (5-6) | 0 | **32** |  |  |  |  |
| EBP-A2 | 5.6 (0.5) | 6 (5-6) | 0 | **60** |  |  |  |  |
| EBP-A3 | 4.8 (0.8) | 5 (5-5) | 0 | **16** |  |  |  |  |
| EBP-A4 | 5.3 (0.7) | 5 (5-6) | 0 | **44** |  |  |  |  |
| EBP-A5 | 5.0 (1.0) | 5 (5-6) | 0 | **32** |  |  |  |  |
| EBP-A6 | 4.2 (0.8) | 4 (4-5) | 0 | 0 |  |  |  |  |
| **Personal application (EBP-P)** | | | | | 16.5 (4.7) | 0 | 0 | 0.874 |
| EBP-P1 | 2.4 (1.0) | 2 (2-3) | **20** | 0 |  |  |  |  |
| EBP-P2 | 3.4 (2.0) | 3 (3-4) | 4 | 12 |  |  |  |  |
| EBP-P3 | 3.2 (1.1) | 3 (3-4) | 8 | 12 |  |  |  |  |
| EBP-P4 | 3.0 (0.9) | 3 (2-3) | 0 | 8 |  |  |  |  |
| EBP-P5 | 2.2(1.0) | 2 (1-3) | **32** | 0 |  |  |  |  |
| EBP-P6 | 2.4 (1.0) | 2 (2-3) | **20** | 0 |  |  |  |  |
| **Future use (EBP-F)** | | | | | 41.4 (5.9) | 0 | 0 | 0.862 |
| EBP-F1 | 5.0 (1.0) | 5 (4-6) | 0 | **36** |  |  |  |  |
| EBP-F2 | 5.2 (0.8) | 5 (5-6) | 0 | **44** |  |  |  |  |
| EBP-F3 | 4.9 (0.8) | 5 (5-5) | 0 | **20** |  |  |  |  |
| EBP-F4 | 5.0 (1.0) | 5 (4-6) | 0 | **40** |  |  |  |  |
| EBP-F5 | 4.9 (0.8) | 5 (4-5) | 0 | **24** |  |  |  |  |
| EBP-F6 | 5.4 (0.9) | 6 (5-6) | 0 | **56** |  |  |  |  |
| EBP-F7 | 4.4 (1.1) | 4 (4-5) | 0 | **20** |  |  |  |  |
| EBP-F8 | 3.3 (1.0) | 4 (3-4) | 4 | 0 |  |  |  |  |
| EBP-F9 | 3.3 (1.1) | 3 (3-4) | 4 | 0 |  |  |  |  |
| **EBP total –  all 26 items** | - | - | - | - | 113.7 (12.3) | 0 | 0 | 0.903 |
| * EBP questionnaire consists of four subscales: knowledge of EBP (EBP-K, 5 items scored on a 6-point Likert scale; score range = 5-30), attitudes toward EBP (EBP-A, 6 items scored on a 6-point Likert scale; score range = 6-36; reverse scored for analysis), personal application and use of EBP (EBP-P, 6 items scored on a 5-point Likert scale; score range = 6-30), and future use of EBP (EBP-F, 9 items scored on a 6-point Likert scale; score range = 9-54). The total score is the sum of these four subscale scores (26-items; score range = 26-150).  **Bolded** floor and ceiling % indicate occurrence of floor or ceiling effects (i.e., ≥15% of participants achieved the lowest or highest possible score. | | | | | | | | |

Item-level and subscale score analysis of the modified EBP questionnaire *after* implementation of a new journal club in chiropractic students and trainees (n=29)

| **Subscale and item** | **Item-level** | | | | **Subscale-level** | | | |
| --- | --- | --- | --- | --- | --- | --- | --- | --- |
|  | **Mean (SD)** | **Median (IQR)** | **Floor %** | **Ceiling %** | **Mean (SD)** | **Floor %** | **Ceiling %** | **Cronbach’s α** |
| **Knowledge (EBP-K)** | | | | | 25.5 (2.9) | 0 | 10 | 0.764 |
| EBP-K1 | 5.3 (0.7) | 5 (5-6) | 0 | **41** |  |  |  |  |
| EBP-K2 | 5.3 (0.8) | 5 (5-6) | 0 | **48** |  |  |  |  |
| EBP-K3 | 5.3 (0.6) | 5 (5-6) | 0 | **34** |  |  |  |  |
| EBP-K4 | 4.9 (0.9) | 5 (4-6) | 0 | **28** |  |  |  |  |
| EBP-K5 | 4.6 (1.0) | 5 (4-5) | 0 | **21** |  |  |  |  |
| **Attitudes (EBP-A)** | | | | | 29.9 (3.1) | 0 | 3 | 0.677 |
| EBP-A1 | 5.0 (0.8) | 5 (5-5) | 0 | **24** |  |  |  |  |
| EBP-A2 | 5.4 (0.6) | 6 (5-6) | 0 | **52** |  |  |  |  |
| EBP-A3 | 5.2 (0.8) | 5 (5-6) | 0 | **38** |  |  |  |  |
| EBP-A4 | 5.3 (0.8) | 5 (5-6) | 0 | **48** |  |  |  |  |
| EBP-A5 | 4.8 (1.0) | 5 (4-6) | 0 | **28** |  |  |  |  |
| EBP-A6 | 4.1 (0.9) | 4 (3-5) | 0 | 7 |  |  |  |  |
| **Personal application (EBP-P)** | | | | | 16.3 (4.6) | 3 | 0 | 0.834 |
| EBP-P1 | 2.6 (0.9) | 2 (2-3) | 7 | 3 |  |  |  |  |
| EBP-P2 | 3.2 (1.0) | 3 (3-4) | 3 | 10 |  |  |  |  |
| EBP-P3 | 3.1 (1.1) | 3 (2-4) | 3 | 10 |  |  |  |  |
| EBP-P4 | 2.7 (1.3) | 2 (2-4) | **17** | 10 |  |  |  |  |
| EBP-P5 | 2.2 (0.9) | 2 (2-3) | **21** | 0 |  |  |  |  |
| EBP-P6 | 2.4 (1.0) | 3 (2-3) | **24** | 0 |  |  |  |  |
| **Future use (EBP-F)** | | | | | 41.1 (6.6) | 0 | 0 | 0.878 |
| EBP-F1 | 4.9 (0.9) | 5 (4-5) | 0 | **24** |  |  |  |  |
| EBP-F2 | 5.2 (0.8) | 5 (5-6) | 0 | **38** |  |  |  |  |
| EBP-F3 | 5.1 (0.8) | 5 (5-6) | 0 | **31** |  |  |  |  |
| EBP-F4 | 5.0 (1.1) | 5 (5-6) | 0 | **38** |  |  |  |  |
| EBP-F5 | 5.0 (0.9) | 5 (4-6) | 0 | **34** |  |  |  |  |
| EBP-F6 | 5.3 (0.8) | 5 (5-6) | 0 | **48** |  |  |  |  |
| EBP-F7 | 4.3 (1.2) | 4 (3-5) | 0 | **17** |  |  |  |  |
| EBP-F8 | 3.4 (1.2) | 4 (3-4) | 7 | 3 |  |  |  |  |
| EBP-F9 | 3.1 (1.2) | 3 (2-4) | 10 | 7 |  |  |  |  |
| **EBP total –  all 26 items** | - | - | - | - | 112.7 (14.6) | 0 | 0 | 0.925 |
| * EBP questionnaire consists of four subscales: knowledge of EBP (EBP-K, 5 items scored on a 6-point Likert scale; score range = 5-30), attitudes toward EBP (EBP-A, 6 items scored on a 6-point Likert scale; score range = 6-36; reverse scored for analysis), personal application and use of EBP (EBP-P, 6 items scored on a 5-point Likert scale; score range = 6-30), and future use of EBP (EBP-F, 9 items scored on a 6-point Likert scale; score range = 9-54). The total score is the sum of these four subscale scores (26-items; score range = 26-150).  **Bolded** floor and ceiling % indicate occurrence of floor or ceiling effects (i.e., ≥15% of participants achieved the lowest or highest possible score. | | | | | | | | |
